# Metformin Improves Ileal Epithelial Barrier Function in Interleukin-10 Deficient Mice

**DOI:** 10.1371/journal.pone.0168670

**Published:** 2016-12-21

**Authors:** Yansong Xue, Hanying Zhang, Xiaofei Sun, Mei-Jun Zhu

**Affiliations:** 1 School of Food Science, Washington State University, Pullman, Washington, United States of America; 2 School of Food Science, University of Idaho, Moscow, Idaho, United States of America; Cincinnati Children's Hospital Medical Center, UNITED STATES

## Abstract

**Background and aims:**

The impairment of intestinal epithelial barrier is the main etiologic factor of inflammatory bowel disease. The proper intestinal epithelial proliferation and differentiation is crucial for maintaining intestinal integrity. Metformin is a common anti-diabetic drug. The objective is to evaluate the protective effects of metformin on ileal epithelial barrier integrity using interleukin-10 deficient (IL10KO) mice.

**Methods:**

Wild-type and IL10KO mice were fed with/without metformin for 6 weeks and then ileum was collected for analyses. The mediatory role of AMP-activated protein kinase (AMPK) was further examined by gain and loss of function study *in vitro*.

**Results:**

Compared to wild-type mice, IL10KO mice had increased proliferation, reduced goblet cell and Paneth cell lineage differentiation in the ileum tissue, which was accompanied with increased crypt expansion. Metformin supplementation mitigated intestinal cell proliferation, restored villus/crypt ratio, increased goblet cell and Paneth cell differentiation and improved barrier function. In addition, metformin supplementation in IL10KO mice suppressed macrophage pro-inflammatory activity as indicated by reduced M1 macrophage abundance and decreased pro-inflammatory cytokine IL-1β, TNF-α and IFN-γ expressions. As a target of metformin, AMPK phosphorylation was enhanced in mice treated with metformin, regardless of mouse genotypes. In correlation, the mRNA level of differentiation regulator including *bmp4*, *bmpr2* and *math1* were also increased in IL10KO mice supplemented with metformin, which likely explains the enhanced epithelial differentiation in IL10KO mice with metformin. Consistently, in Caco-2 cells, metformin promoted claudin-3 and E-cadherin assembly and mitigated TNF-α-induced fragmentation of tight junction proteins. Gain and loss of function assay also demonstrated AMPK was correlated with epithelial differentiation and proliferation.

**Conclusions:**

Metformin supplementation promotes secretory cell lineage differentiation, suppresses inflammation and improves epithelial barrier function in IL10KO mice likely through activation of AMPK, showing its beneficial effects on gut epithelial.

## Introduction

Inflammatory bowel disease (IBD) including ulcerative colitis (UC) and Crohn's disease (CD) is an inflammatory autoimmune disease commonly occurring in the intestine, and up to now, has no medical treatments with ideal outcomes. Accumulating evidence shows that impairment in intestinal epithelial barrier is highly correlated with IBD and related gastrointestinal diseases [1, 2]. Interleukin-10 (IL-10) is an anti-inflammatory cytokine that inhibits macrophage activation and inflammatory response [3, 4]. The expression of IL-10 in IBD patients was lower than that in normal people [5]. As a result, IL-10 deficient or IL10 knockout (IL10KO) in mouse induces chronic gut inflammation and develops a disease resembling CD with ileum inflammation [6]. Thus, IL10KO mouse is one of the most common models for studying IBD [7].

The small intestinal epithelium constitutes crypts and villi. Newly generated proliferating cells from intestinal stem cells either migrate to the tip of villi and terminally differentiate into goblet cells, enterocytes or enteroendocrine cells [8], or alternatively descent to the bottom of crypts to differentiate into Paneth cells [9, 10]. Paneth cells secret antimicrobial peptides and lysozyme, critical for defending against intestinal microbiota, while goblet cells secrete mucins to repel antigens and maintain intestinal homeostasis [9]. Dysregulation of intestinal proliferation and differentiation underpins many intestinal immunological diseases including IBD.

Metformin, a widely used antidiabetic drug [11, 12], has protective roles in cardiovascular complications [13], and anti-inflammatory effects on vascular endothelial cells by preventing nuclear factor κB (NF-κB) activation and attenuating TNF-α induced inflammatory response [14, 15]. A recent study revealed that metformin attenuated colonic inflammation in both DSS-induced wild-type (WT) mice and IL10KO mice [16], suggesting the protective role of metformin in gut epithelium. However, the underlying mechanisms have not been explored. Notch signaling is known to be critical for epithelial differentiation, and it promotes *math1* expression [17]; and bone morphogenetic proteins (BMPs) are required for proper epithelial differentiation by interacting with transcription factors such as Math1 [18]. Interestingly, in osteoblastic MC3T3-E1 cells, metformin induced endothelial BMP2 expression via AMPK activation [19, 20]. Binding of BMPs to their receptors induces the recruitment and phosphorylation of Smads, which then regulates gene expression and cell differentiation [21]. We hypothesized that metformin enhances gut epithelial differentiation and barrier function partially through activating AMPK. Since orally administered metformin is selectively accumulated in small intestine [22], where IBD commonly develops, this study evaluated the potential protective role of metformin in ileal epithelial barrier function using IL10KO mice, the commonly used mouse model of IBD.

## Materials and Methods

### Mice and experimental procedures

All animal studies were conducted in accordance with the protocols approved by the Animal Use and Care Committee of Washington State University. IL10KO male mice were purchased from Jackson Lab (Bar Harbor, ME). C57BL/6 (WT) and IL10KO mice were raised under specific pathogen-free conditions with controlled temperature and light [23]. Six-week-old IL10KO and WT mice were fed with regular water (CON) or water containing metformin (0.2% w/v) for 6 weeks, which resulted in four treatment groups; WT-CON (n = 10), WT-MET (n = 10), IL10KO-CON (n = 10), and IL10KO-MET (n = 10). During the trial, water was changed every other day; feed intake and body weight were monitored weekly. All mice were given feeds and water *ad libitum*.

### Ileal sample collection

Mice sacrificing and ileal sample collection were described as before [24]. Briefly, mice were anesthetized and sacrificed by cervical dislocation. The terminal ileums with 5 mm segment were fixed in 4% paraformaldehyde for histological analysis. The remaining ileal tissues were stored at -80°C.

### Intestinal permeability assay

Intestinal permeability was conducted as previously published method [23] with modifications. One week before necropsy, mice were fasted for 5 hours and then gavaged with FITC-dextran (500mg/kg body wt). One hour post-gavage, mice blood was collected and centrifuged. The serum was diluted with PBS at a ratio of 1:5 (v/v) and measured the fluorescence intensity at an excitation 485 nm and emission 520 nm with a Synergy H1 Hybrid Multi-Mode Microplate Reader (BioTek Instruments,Winooski, VT, USA). Due to the age gap between IL10KO and WT mice, we conducted intestinal permeability tests of these two genotypes in two separate times.

### *In vivo* stem cell proliferation

IL10KO mice under treatments were injected with 100 μl of BrdU (10 mg/ml in saline, ip, Sigma), a nucleotide analog incorporating into the DNA of proliferating cells, 2 h prior to sacrifice to label S-phase cells. Following sacrifice, a small section of ileum was fixed and paraffin embedded for evaluating BrdU positive cells by immunohistochemical (IHC) staining.

### Immunoblotting analysis

Immunoblotting analyses were performed as published method [23]. Briefly, protein samples were extracted from mice ileum tissues and separated with 4–20% SDS-PAGE followed with nitrocellulose membrane transferring for immunoblotting analyses. Primary antibodies against E-cadherin, p-AMPKα, total AMPKα, p-Acetyl CoA carboxylase (ACC) and PCNA were obtained from Cell Signaling (Beverly, MA). p-Smad1/5/8 was purchased from Santa Cruz (Santa Cruz, CA) and claudin-3 was from Invitrogen (Waltham, MA). Primary antibody against β-actin was from Sigma-Aldrich. Band density was normalized with the β-actin density.

### RNA extraction and quantitative real time PCR analysis

The ileal total RNA was isolated with Trizol (Sigma), followed with DNase I (Qiagen, Valencia, CA) treatment and RNeasy^®^ Mini Kit (Qiagen) purification. cDNA was synthesized using the iScript^™^ kit (Bio-Rad). qRT-PCR was carried out on a CFX96 thermocycler (Bio-Rad) with Sybr Green (Bio-Rad) by using β-actin as the housekeeping gene [23]. Primers used in this study are listed in S1 and S2 Tables.

### IHC staining

IHC staining was conducted as previously described [25]. Briefly, deparaffinized and rehydrated ileum sections were incubated in citrate acid buffer at 95°C for 15 min, blocked in 1.5% goat serum for 30 min and then stained with Ki67 (cell signaling, Beverly, MA), BrdU (cell signaling, Beverly, MA) or lysozyme antibody (Thermo Scientific, Waltham, MA) at 4°C overnight. After incubation, tissue sections were rinsed with PBST, stained with secondary antibodies for 30 min, followed by the Vectastain ABC incubation (Vector Lab, Burlingame, CA) and DAB (Vector Lab) staining. Histological examination was conducted by using a Leica DM2000 microscope (Chicago, IL). The number of Ki67, BrdU or lysozyme positive cells was counted for each crypt in a randomly selected view. The average number was calculated and analyzed (n = 10; 15 crypts each).

M1 macrophage IHC staining was conducted as described above by using anti-mouse iNOS antibody purchased from Invitrogen (Waltham, MA). The M1 positive macrophage scoring system was described previously with modifications [26]. The system used a 0–5 score scale according to the density of macrophage infiltration area: 0 = rare; 1 = 5–10% positive staining with minor infiltration; 2 = 10–30% positive staining; 3 = 30–50% positive staining; 4 = 50–70% positive staining, 5 = >70% positive staining with intense infiltration. The mean score of at least five sections per animal was calculated as M1 score.

### Histological measurement and goblet cell staining

Ileum tissues were paraffin embedded and sectioned (4–5 μm thick). Deparaffinized and rehydrated ileum sections were stained with hemotoxylin and eosin Y (H&E). Images were captured by a Leica LED light microscope (DM2000, Chicago, IL). The measurement of villus and crypt was determined with the Image J 1.30v software. Five sections at constant interval (50 μm) and five fields per section were randomly selected for taking images [23].

For goblet cells staining, deparaffinized and rehydrated ileum section were stained with Alcian blue (pH2.5). Five randomly picked fields per section were quantified for the ratio of goblet cells/villus using the Image J 1.30v software [23].

### AMP and ATP content of ileum tissues measured by HPLC analysis

Ileum tissues were homogenized in 0.9 N HClO_4_ and stayed on ice. After 30 min, centrifuged the lysates and collected the supernatants. The supernatants were neutralized with 2 M KOH and then centrifuged again. Final supernatants were filtered with 0.45μm PTFE filter and injected into Shimadzu HPLC system (Kyoto, JAPAN) equipped with Luna C18 (2) column purchased from Phenomenex (Torrance, CA, USA) as previously described method [27]. Amounts were analyzed using peak areas and peak identification was confirmed using ATP (Sigma, St. Louis, MO) and AMP (Sigma, St. Louis, MO) standards.

### Caco-2 cell treatment and transfection

Caco-2 cell line purchased from American Type Culture Collection (Manassas, VA) was grown at 37°C with 5% CO_2_ in DMEM (Sigma) supplemented with 10% fetal bovine serum (GE, Fairfield, CT) and 1% penicillin-streptomycin (Life Technologies, Grand Island, NY). For metformin treatment, the Caco-2 cells were cultured in a 12-well plate until confluence. The cells were then treated with 1 mM metformin for 5 days. For transfection, Caco-2 cells at 70% confluence were transfected with plasmid constructs containing pAMPKα WT (WT), pAMPKα K45R mutant (K45R), or green fluorescent protein (EGFP) (CON) (Addgene, Cambridge, MA; catalog no. 15991, 15992 and 13031) using Lipofectamine 3000 (Life Technologies) per manufacturer’s instructions. Medium was changed 12 h post transfection, when 400 μg/ml G418 (Amresco, Solon, OH) was added to the transfected cells in the following 7 days to select cells with transfection. The transfected cells were then seeded onto 12-well plates at 2×10^5^ cells per well for 4 days, when cells were collected for RNA extraction and protein sample preparation.

### Calcium switch assay

The calcium switch assay was conducted as previously described with modifications [28]. Briefly, Caco-2 cells were cultured in a 24-well plate until confluence, then treated with calcium free DMEM complete medium by adding 4mM EGTA for 30 min. After washing 3 times with PBS, the cells were switched back to regular DMEM complete medium supplemented with or without 1 mM metformin and incubated for 12 h. The Caco-2 cells treated with EGTA did not switch back to regular DMEM complete medium was used as negative control. The cells were then subjected to immunofluorescence microscopy assay.

### Immunofluorescence microscopy

The Caco-2 cells were cultured in a 24-well plate until confluence. The cells were treated with 0, 10 ng/ml TNF-α or 10 ng/ml TNF-α and 1mM metformin in DMEM complete medium for 24 h. Then the cells were wash 3 times with PBS and fixed in ice-cold methanol for 15 min. After washing with PBS, the fixed cells were blocked with 5% normal goat serum at room temperature for 60 min. Then the cells were incubated with anti-claudin-3 (1:200) and anti-E-cadherin (1:200) at 4°C overnight. After incubation, the stained cells were rinsed with PBS, stained with goat anti-rabbit Alexa Fluor 555 (Beverly, MA) for 60 min and mounted with DAPI (Vector Lab, Burlingame, CA). Fluorescence was visualized with EVOS FL fluorescence microscope (Life Technologies) [29]. The fluorescence intensities of claudin-3 and E-cadherin at the intercellular junctions were measured with Image J. The cell fluorescence intensity is calculated as the following equation: Cell fluorescence = Integrated fluorescence density—Gray value (area of the selected cell × mean fluorescence of intracellular background reading). Fluorescence intensities of twenty randomly picked cells from each section were quantified. The data were expressed as relative fluorescence density per cell.

### Statistical analyses

Statistical data analyses were carried out using General Linear Model of Statistical Analysis System as described in our previous publication [23], and presented as Mean ± standard errors of mean (SEM). A *P* value less than 0.05 is considered as significant [23].

## Results

### Metformin improves gut epithelial barrier function

**Fig 1:**
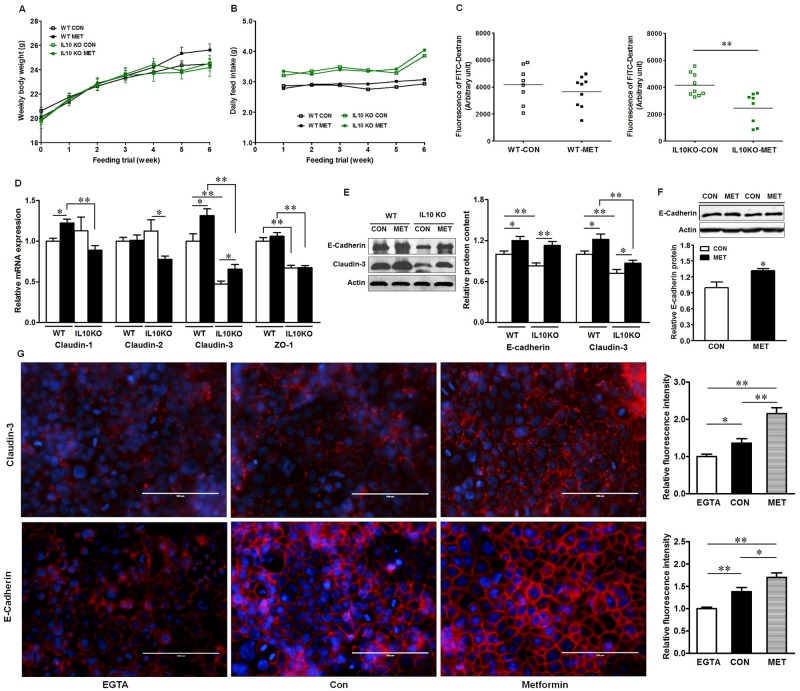
Metformin improves intestinal epithelial barrier function *in vivo* and *in vitro*. (A) Weekly body weight; (B) Daily feed intake; (C) *In vivo* intestinal permeability assay; (D) The mRNA levels of tight junction proteins in mouse ileum tissues; (E) Immunoblotting of claudin-3 and E-cadherin in mouse ileum tissues; (F) Immunoblotting of E-cadherin in Caco-2 cells treated with or without metformin. (G) Immunofluorescent staining of claudin-3 and E-cadherin in Caco-2 cells with calcium switch. The cells were subjected to calcium depletion for 30 min followed by incubation in complete medium for 12 h supplemented with or without 1mM metformin. EGTA: Caco-2 cells with calcium depletion; Con: Caco-2 cells were Ca^2+^ restored and treated without metformin; Metformin: Caco-2 cells were Ca^2+^ restored and treated with metformin. Scale bar is 100μm. (*: *P* < 0.05; **: *P* < 0.01; means ± SEM, n = 10).

Metformin supplementation did not affect body weight gain (*P* >0.05) (Fig 1A) and feed intake (*P* >0.05) (Fig 1B). Interestingly, IL10KO mice had higher feed intake than WT mice throughout feeding trial (Fig 1B) (*P* <0.05). Metformin supplementation decreased intestinal permeability in IL10KO mice (*P* <0.01) (Fig 1C), indicating improved barrier function. However, no change was observed in WT mice with/without metformin supplementation. The intestinal permeability between genotypes was not compared since these two groups were conducted in two separated time points. To further determine the mechanisms leading to the improved barrier function in IL10KO mice, we analyzed the expressions of tight junction proteins (Fig 1D). Claudin-2 is considered as a pore-forming tight junction protein that increases epithelial permeability in active Crohn's disease [30]. The mRNA level of claudin-2 was not changed in WT mice with/without supplementation, while its expression was reduced in IL10KO mice due to metformin treatment (*P* <0.05). Metformin supplementation had no effect on the expression of ZO-1, a peripheral scaffolding protein that seals the adjacent cells [31, 32], but IL-10 ablation reduced ZO-1 expression (*P* <0.01). Claudin-3, another critical component of tight junction complex which is constantly expressed in healthy gut and promotes intestinal barrier function [33], was also reduced in IL10KO mice (*P* <0.01), which was recovered by metformin supplementation (*P* <0.05) (Fig 1D and 1E). Consistently, E-cadherin, a core component of the adhesion junctions that is required for the maintenance of intestinal epithelial integrity [34], was decreased in IL10KO mice as compared with that of WT mice (*P* <0.05) (Fig 1E), which were enhanced (*P* < 0.05) by metformin supplementation in both genotypes and was further demonstrated in Caco-2 cells (Fig 1E and 1F). Using calcium switch assay and immunofluorescent staining, we found that in low calcium state, claudin-3 and E-cadherin were distributed sparsely and diffusely. However, metformin treatment accelerated and enhanced claudin-3 and E-cadherin reassembly after tight junction destruction (Fig 1G). Collectively, our data demonstrated metformin improved intestinal epithelial barrier function.

**Fig 2:**
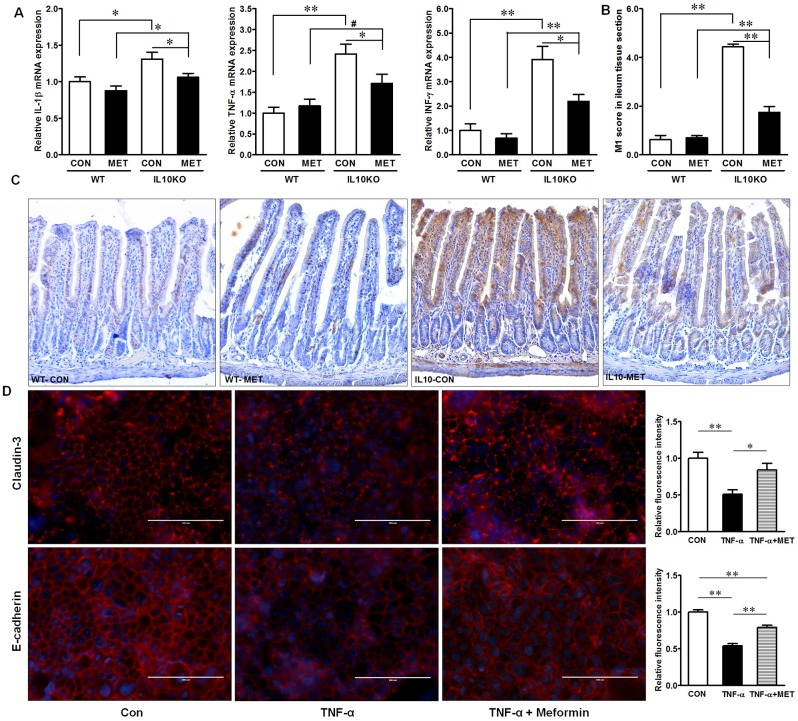
Inflammatory response in IL10KO or WT mice supplemented with (■, MET) or without (□, CON) metformin. (A) Inflammatory cytokine mRNA expression; (B) and (C) M1 macrophage IHC staining; (D) Immunofluorescent staining of claudin-3 and E-cadherin in Caco-2 cells treated with TNF-α and with/without co-incubation with metformin for 24 h. Con: Caco-2 cells without treatment; TNF-α: Caco-2 cells treated with 10ng/ml TNF-α; TNF-α + Metformin: Caco-2 cells treated with 10ng/ml TNF-α and 1mM metformin. Scale bar is 100μm. (#: *P* < 0.1; *: *P* < 0.05; **: *P* < 0.01; means ± SEM; n = 10).

Gut inflammation is known to impair epithelial barrier function. Consistent with barrier changes, the mRNA expression of key pro-inflammatory cytokines, IL-1β, TNF-α and IFN-γ were dramatically increased in IL10KO mice as compared with WT-CON mice (Fig 2A). Metformin supplementation mitigated pro-inflammatory cytokine expressions in IL10KO mice, but not in WT mice (Fig 2A). In agreement, M1 proinflammatory macrophage was more abundant in IL10KO mice as compared with that in WT mice (Fig 2B and 2C). Metformin supplementation significantly attenuated M1 macrophage infiltration in IL10KO mice (Fig 2B and 2C). TNF-α is mainly produced by pro-inflammatory macrophages and is a potential contributing factor to IBD [35]. In Caco-2 cells, TNF-α treatment impaired claudin-3 and E-cadherin assembly, which were mitigated by metformin treatment (Fig 2D). These indicated that metformin had beneficial effects on mitigating inflammation and inflammation induced barrier destruction.

### Metformin suppresses proliferation and promotes differentiation of epithelial cells in IL10KO mice

**Fig 3:**
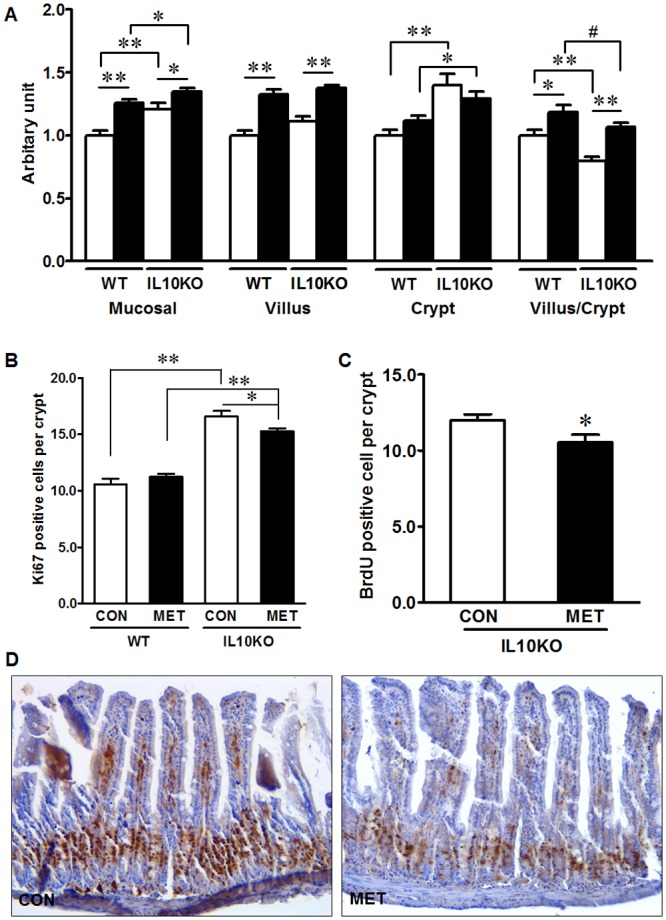
Morphological alteration and epithelial proliferation markers in IL10KO or WT mice supplemented with (■, MET) or without (□, CON) metformin. (A) Ileum morphological analysis; (B) Proliferative cells identified by Ki67 IHC staining; (C) and (D) Proliferative cells identified by BrdU incorporation. Images were taken at 200× magnification. (#: *P* < 0.1; *: *P* < 0.05; **: *P* < 0.01; means ± SEM; n = 10).

Histological analysis of ileum tissue showed that both WT and IL10KO mice had higher villus length when supplemented with metformin (*P* <0.01) (Fig 3A). However, metformin supplementation did not alter crypt depth, resulting in an overall increase in mucosal thickness of both WT and IL10KO ileum. On the other hand, IL-10 depletion elongated crypt depth (*P* <0.01) and increased mucosal thickness with no change in villus length, resulting in decreased villus/crypt ratio, which was restored by metformin supplementation (Fig 3A).

To gain more insights on the altered ileum morphologies, we further analyzed the proliferation of crypt cells. Consistent with the increased crypt depth, the proliferation as assessed by Ki67 staining in IL10KO mice was enhanced (*P* < 0.01) (Fig 3B). Though the crypt depth was only numerically decreased in IL10KO mice supplemented with metformin compared with IL10KO CON mice, metformin decreased the number of proliferative cells per crypt in IL10KO mice (Fig 3B). Metformin had no effect on the proliferative cell density in WT mice, which is in agreement with the unchanged crypt depth. *In vivo* BrdU labeling further revealed that the percentage of BrdU-positive cells was lower in metformin treated IL10KO mice as compared with IL10KO CON mice (*P* <0.05) (Fig 3C and 3D). In combination, data showed that IL10 deficiency increased cell proliferation, which was mitigated due to metformin supplementation.

**Fig 4:**
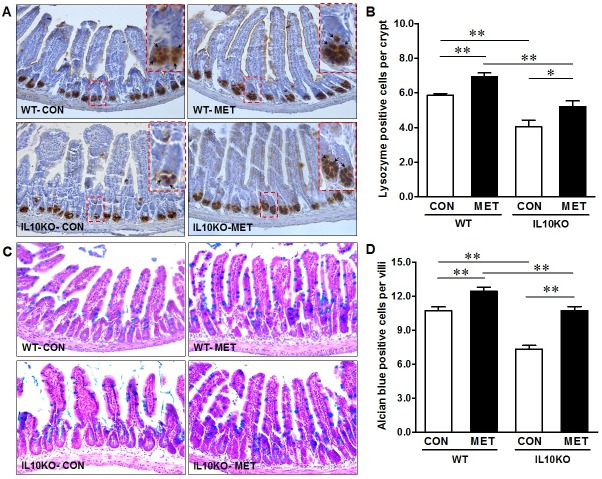
IHC staining for Paneth cell and goblet cell in IL10KO or WT mice supplemented with (■, MET) or without (□, CON) metformin. (A) and (B) Lysozyme positive Paneth cells; (C) and (D) Goblet cell density. Images were taken at 200× magnification. (*: *P* < 0.05; **: *P* < 0.01; means ± SEM; n = 10).

Hyperproliferation is commonly correlated with impaired differentiation, which was further examined. Paneth cells locate in the crypts of Lieberkuhn, secreting a variety of products such as lysozyme, antimicrobial peptides, growth factors, and phospholipase A2 [36]. Using lysozyme as a marker, we found the Paneth cell density was decreased dramatically in IL10KO mice (*P* <0.01) (Fig 4A and 4B); metformin treatment increased Paneth cells number in IL10 KO mice as well as in WT mice (Fig 4A and 4B). Consistently, Alcian blue staining revealed that the number of goblet cells was much lower in the ileum of IL10KO mice as compared with that of WT mice (Fig 4C and 4D), while metformin supplementation increased (*P* <0.01) the goblet cell number in both IL10KO and WT mice. In aggregate, IL-10 depletion induced excessive proliferation of epithelial cells in the ileum gut, which was associated with impaired differentiation of the Paneth cell and goblet cell lineages. Metformin supplementation recovered these adverse changes observed in IL10KO mice.

### Metformin enhances the expression of key transcription factors and promotes epithelial differentiation

**Fig 5:**
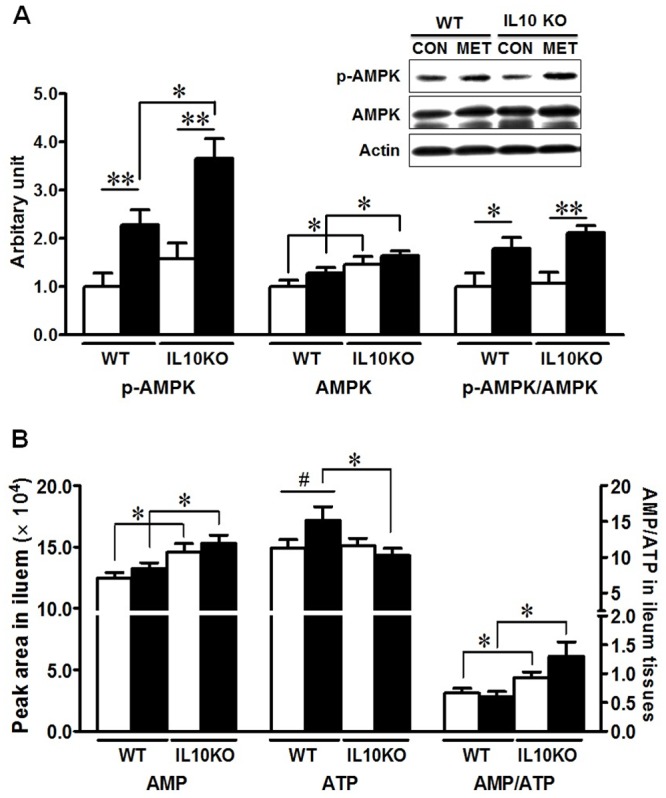
AMPK signaling in IL10KO or WT mice supplemented with (■, MET) or without (□, CON) metformin. (A) AMPK and p-AMPK analyzed by immunoblotting. (B) AMP and ATP content in ileum tissues analyzed by HPLC. (#: *P* < 0.1; *: *P* < 0.05; **: *P* < 0.01; means ± SEM; n = 10).

AMPK is known to promote cell differentiation [37]. We further analyzed AMPKα phosphorylation at Thr 172, which correlates with AMPK activity. As shown in Fig 5A, metformin stimulated AMPK phosphorylation (*P* <0.01), regardless of mouse genotypes (Fig 5A). Since AMPK activity is regulated by cellular AMP/ATP ratio, the ileal contents of ATP and AMP were further measured by HPLC. IL10KO mice had a higher AMP and AMP/ATP ratio as compared with WT mice regardless of metformin supplementation (Fig 5B). Metformin supplementation did not affect AMP/ATP ratio within genotypes (Fig 5B).

**Fig 6:**
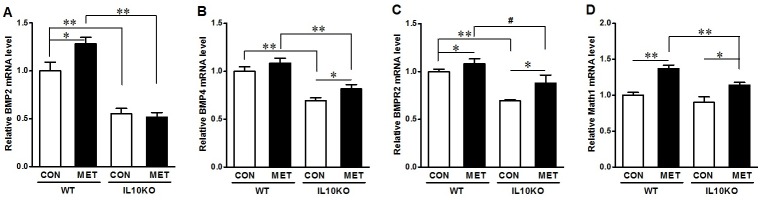
mRNA expression of key mediators of BMP signaling and transcription factors regulating epithelial differentiation in IL10KO or WT mice supplemented with (■, MET) or without (□, CON) metformin. (A) *bmp2*; (B) *bmp4*; (C) *bmpr2*; D. *math1*. (#: *P* < 0.1; *: *P* < 0.05; **: *P* < 0.01; means ± SEM; n = 10).

To identify the mechanisms leading to the enhanced epithelial differentiation, we analyzed the factors regulating epithelial differentiation. Metformin enhanced *bmp2* expression in WT mice and *bmp4* in IL10KO mice (Fig 6A and 6B). However, the expression of *bmpr2* was reduced in IL10KO mice without metformin treatment as compared with WT mice, which was prevented due to metformin supplementation (Fig 6C). Consistently, the expression of *math1* was also enhanced due to metformin treatment in both WT and IL10KO mice (Fig 6D).

**Fig 7:**
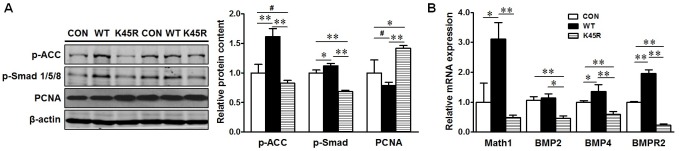
AMPK is associated with the mediators of intestinal epithelial cell differentiation and proliferation *in vitro*. Caco-2 cells were transfected with plasmid constructs over-expressing green fluorescent protein (EGFP) (CON), wild-type AMPK (WT) or dominant negative AMPK (K45R), and harvested for mRNA and protein measurement 4 days later. (A) Immunoblotting of p-ACC, p-Smad1/5/8 and PCNA; Left panel showed the representative bands of western blotting; Right panel showed the statistical analysis for each transfected clone. (B) mRNA expression of *math1*, *bmp2*, *bmp4* and *bmpr2*. #: *P* < 0.1; *: *P* < 0.05; **: *P* < 0.01; means ± SEM; n = 4).

To examine the potential mediating role of AMPK in linking beneficial effects of metformin to epithelial differentiation, we used the over-expression of plasmids carrying AMPKα WT or K45R which is kinase dead to alter AMPK activity in the cultured Caco-2 cells. As expected, AMPKα WT over-expression activated AMPK signaling as shown by enhanced ACC phosphorylation, which is exclusively phosphorylated by AMPK, while AMPK K45R reduced AMPK activity (Fig 7A). The phosphorylation of Smad1/5/8, which is a down-stream effector of BMP signaling and phosphorylated by BMP receptors, was correlated with AMPK activity (Fig 7A). Consistently, the level of *math1*, *bmp4* and *bmpr2* mRNA expression was also increased by AMPK activation and reduced due to AMPK inhibition (Fig 7B). In contrast, the PCNA content was decreased in AMPK WT group while increased in K45R expressing cells (Fig 7A). These data showed that AMPK, the molecular target of metformin, might promote the expression of transcription factors that promoting epithelial differentiation.

## Discussion

Intestinal epithelium constitutes a single layer of tightly linked cells that are constantly and actively renewed throughout lifespan [38]. The balance among proliferation, migration, and differentiation is tightly controlled and regulated, which is vital to intestinal homeostasis. Excessive proliferation results in enlarged crypt size [39]. Dextran sulfate sodium (DSS) treated rat showed a greater proliferation rate and crypt depth in small intestine [40]. Consistently, in current study, we found that IL10KO mice had enhanced cell proliferation, increased crypt length and decreased villus/crypt ratio in ileum, while metformin supplementation decreased the number of proliferative cells and restored villus/crypt ratio in IL10KO mice.

Inflammation enhances intestinal proliferation [41]. Excessive inflammatory response leads to abnormal epithelial morphology with prolonged crypts, excessive proliferation, and even neoplasia [42]. Inflammation also disrupts intestinal epithelial barrier function by decreasing barrier forming tight junction proteins like ZO-1 and increasing the pore-forming protein such as claudin-2, which increases transepithelial permeability [43]. It has been reported that claudin-3 is critical for the function of tight junctions while inflammation could damage the distribution of claudin-3 [44]; a reduced level of claudin-3 was observed in active IBD patients [30], which is consistent with our study that inflammation diminished claudin-3 both *in vitro* and *in vivo*. On the other hand, overexpression of claudin-3 increased transepithelial resistance (TER) and improved barrier function [44]. In agreement, our data revealed that metformin treatment could restore claudin-3 and E-cadherin assembly impaired by calcium switch and inflammation. At the same time, enhanced permeability and impaired tight junction allow antigens to enter into underlying tissues that cause exaggerated immune responses and severe intestinal inflammation [45]. Consistent with previous observation, IL10KO mice had enhanced inflammation and increased cell proliferation in ileum. This was mitigated by metformin supplementation, in accordance with improved barrier function in IL10KO mice. Our finding was further supported by a recent publication in colonic epithelial cells, where metformin ameliorated colitis severity in DSS-induced mice associated with attenuated NF-κB inflammatory signaling in the distal colonic epithelium, and mitigated inflammatory response in the proximal colon of IL10KO mice [16]. On the other hand, the barrier function was improved due to metformin-induced AMPK activation, which is demonstrated in our previous study that AMPK activation enhanced TER and decreased paracellular permeability in Caco-2 cells by gain and loss function assays [46]. By silencing AMPK with AMPKα K45R (K45R) plasmid, the transfected Caco-2 cells had much lower TER and higher permeability as compared with control and AMPK overexpression cells [46]. In addition, AMPK knockout mice had a higher intestinal permeability when compared with WT mice [46].

Macrophage plays an essential role in maintaining intestinal homeostasis, which can be classified as “classical activation” expressing Th1 inflammatory cytokine such as TNF-α and IFNγ and “alternative activation” with Th2 cytokines IL-4 and IL-13 production [47]. In the disease status, macrophages produce a diverse repertoire of pro-inflammatory mediators inducing an inflammatory environment in the gut. Metformin is a potent AMPK activator, and AMPK activation helps to induce macrophage polarization into an anti-inflammatory phenotype [48]. In addition, metformin regulates the balance between Th17 and T_reg_ cells [49]. Treatment of metformin helps the differentiation of T_reg_ cells while down-regulating proinflammatory Th17 cells, which subsequently ameliorates inflammation in inflamed bowel [49]. In current study, metformin decreased IFN-γ and TNF-α contents, and reduced the recruitment of pro-inflammatory macrophages, suggesting that metformin/AMPK could ameliorate inflammation and associated epithelial barrier damage. However, inflammation could also inhibit AMPK activity through stimulating AMPK de-phosphorylation [50]. We found a higher AMP/ATP ratio in IL10KO mice, which is consistent with a previous study showing that inflamed CD ileum had a lower ATP level [51]. The combined effects of AMPK de-phosphorylation due to inflammation might neutralize the effect of high AMP/ATP, explaining the lack of difference in AMPK phosphorylation between genotypes.

One of the most manifested characteristics of IBD is the imbalance between epithelial cell proliferation and differentiation. Excessive proliferation and limited differentiation impair intestinal epithelial function, which causes various intestinal diseases including cancer [52]. Well-differentiated epithelial cells enhance the contents of barrier forming proteins and antigen presentation [53, 54], while deficiency in epithelial differentiation is known to impair barrier function [55, 56]. Goblet cells and Paneth cells are two critical differentiated cells that maintain normal barrier function in small intestine, while IBD subjects exhibit defective cell differentiation and function [57]. Goblet cells secrete mucins, which form intestinal mucosa that protects mucus layer from transmission of harmful antigens and pathogens [58]. Defective goblet cells are associated with the symptoms of colitis and the severity of IBD [59], which could be due to the loss of goblet cells/mucin-2 [60, 61]. Paneth cells has a central role in maintaining the stem cell compartment in small intestine through secreting versatile signals, growth factors and antimicrobial peptides. Ileal CD disorder is characterized by decreased secretion of antimicrobial α-defensins by Paneth cells [62], and low expression of β-defensin 2 [63]. Dysfunction of Paneth cells is an intrinsic factor responsible for defects in immune response to pathogen-induced inflammation in CD [64]. Consistently, we found that both goblet cells and Paneth cells were reduced in IL10KO as compared with WT mice, which were restored by metformin supplementation. These changes likely explain the beneficial effects of metformin on gut epithelial differentiation and protective function.

To further define the mechanisms, we analyzed mediators regulating epithelial differentiation. Recent studies show that AMPK promotes the differentiation of stem cells in a number of tissues, including gut epithelium [37, 65]. Our previous study demonstrated that AMPK activation enhanced gut epithelial differentiation while AMPK knockout mice have impaired intestinal differentiation associated with aggravated intestinal permeability [46]. BMP signaling is one of the key pathways regulating gastrointestinal development and homeostasis [66, 67], known to promote epithelial differentiation [18], and maintain ileal morphogenesis [20]. BMP2 and BMP4 elicit function and activate down-stream signaling by binding to their type II serine/threonine receptor (BMPR2) and further dimerizing with type I receptors (BMPR1A or BMPR1B) [66]. We found that metformin supplementation enhanced BMP signaling in addition to AMPK activation in IL10KO mice, which is in agreement with a previous report showing the activation of BMP signaling by AMPK in bone cells [19]. AMPK induces BMP2 expression [19], which binds to its receptors that activates Smad signaling in IL10 KO mice treated with metformin. Our data are consistent with a previous report showing that phosphorylation of Smad1/5/8 is increased by AMPK activation in osteoblasts which correlates with osteogenic differentiation [68]. Aligned with changes in BMP signaling, the expression of key transcription factor promoting epithelial differentiation, Math1 was also promoted by metformin.

Because metformin activates AMPK, to conclusively establish the role of AMPK in mediating beneficial effects of metformin on epithelial differentiation, we further conducted AMPK gain and loss of function study in Caco-2 cells. We found that AMPK activity is correlated with the expression of factors regulating epithelial differentiation, which is consistent with our finding that AMPK induces differentiation [46]. In combination, metformin promoted epithelial differentiation likely via activation of AMPK.

**Fig 8:**
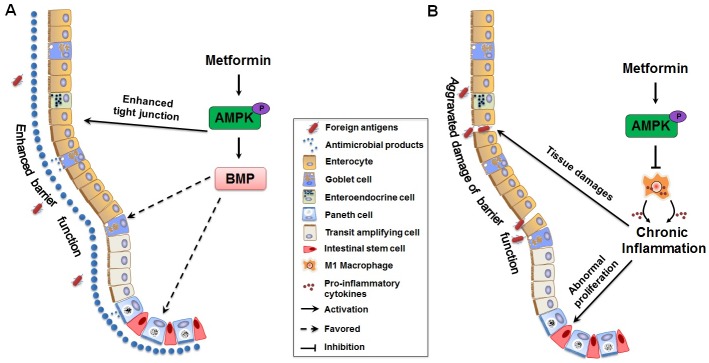
Model for effects of metformin on ileal epithelial barrier function. (A) Metformin supplementation induce differentiation of goblet cells and Paneth cells through the activation of AMPK-mediated BMP signaling pathway. The differentiated cells secrete mucins and anti-microbial components that compose the mucus layer (indicated as big blue dots), which enhances epithelial barrier function and thus defense against foreign antigens (indicated as red solid ovals) (B) Chronic inflammation induced by activated pro-inflammatory macrophages destructs epithelial integrity and causes intestinal epithelial abnormal proliferation. Metformin supplementation attenuates chronic inflammatory response and restores barrier function through the activation of AMPK.

In conclusion, our study provided evidences that metformin improved gut epithelial health through promoting secreted cell differentiation and inhibiting intestinal proliferation and inflammation in IL10KO mice, which were potentially associated with activated AMPK and enhanced expression of key factors promoting epithelial differentiation (Fig 8).

## Supporting Information

S1 Table Primer sets used for quantitative RT-PCR in mouse tissues. (DOCX)

S2 Table Primer sets used for quantitative RT-PCR in human cells. (DOCX)
