# Supplementary material for: Metformin Improves Ileal Epithelial Barrier Function in Interleukin-10 Deficient Mice
Source: PLoS One. 2016 Dec 21;11(12):e0168670. doi: 10.1371/journal.pone.0168670 (PMC5176295; doi:10.1371/journal.pone.0168670)
Supplement: S1 Table — (DOCX) [file pone.0168670.s001.docx]

**S1 Table. Primer sets used for quantitative RT-PCR in mouse tissues**

| **Gene Name** | **Accession No.** | **Product Size** | **Direction** | **Sequence (5’🡪3’)** | **Source** |
| --- | --- | --- | --- | --- | --- |
| **β-actin** | [NM_007393.3](http://www.ncbi.nlm.nih.gov/nucleotide/145966868?report=gbwithparts) | 183bp | Forward | GATCAAGATCATTGCTCCTCCTG | This study |
|  |  |  | Reverse | AGGGTGTAAAACGCAGCTCA |  |
| **BMP2** | NM_007553.3 | 83bp | Forward | TGCTTCTTAGACGGACTGCG | [1] |
|  |  |  | Reverse | CTGGGGAAGCAGCAACACTA |  |
| **BMP4** | NM_007554.2 | 105bp | Forward | CCCGGAAGCTAGGTGAGTTC | This study |
|  |  |  | Reverse | AATCCCATCAGGGACGGAGA |  |
| **BMPR2** | NM_007561.4 | 241bp | Forward | GCAGCAGTATACAGATAGGTGAGT | This study |
|  |  |  | Reverse | TTAGACACTGTGGTCGTGGC |  |
| **Claudin-1** | NM_016674.4 | 80bp | Forward | AGGAGGGAAGGCTTTTGCCTGTGA | [2] |
|  |  |  | Reverse | ATGCAAGGAGCACCTTATCCCCGT |  |
| **Claudin-2** | NM_016675.4 | 120bp | Forward | GGCGTCCAACTGGTGGGCTAC | [2] |
|  |  |  | Reverse | AACCGCCGTCACAATGCTGGC |  |
| **Claudin-3** | NM_009902.4 | 132bp | Forward | CAGGGGCAGTCTCTGTGCGAG | [2] |
|  |  |  | Reverse | GCCGCTGGACCTGGGAATCAAC |  |
| **Math1** | NM_007500.4 | 108bp | Forward | GTGCGATCTCCGAGTGAGAG | [1] |
|  |  |  | Reverse | GGGATAAGCCCCGAACAACA |  |
| **IFN-γ** | NM_008337.3 | 93bp | Forward | AGGTCCAGCGCCAAGCATTCAA | [2] |
|  |  |  | Reverse | AGCAGCGACTCCTTTTCCGCTT |  |
| **IL-1β** | M15131 | 152bp | Forward | CAACCAACAAGTGATATTCTCCATG | [3] |
|  |  |  | Reverse | GATCCACACTCTCCAGCTGCA |  |
| **TNF-α** | NM_013693.2 | 67bp | Forward | TGGGACAGTGACCTGGACTGT | [4] |
|  |  |  | Reverse | TTCGGAAAGCCCATTTGAGT |  |
| **ZO-1** | NM_009386.2 | 403bp | Forward | ACCCGAAACTGATGCTGTGGATAG | [4] |
|  |  |  | Reverse | AAATGGCCGGGCAGAACTTGTGTA |  |

**References**

1. Yang G, Xue Y, Zhang H, Du M, Zhu MJ. Favourable effects of grape seed extract on intestinal epithelial differentiation and barrier function in IL10-deficient mice. Br J Nutr. 2015;114(1):15-23. doi: 10.1017/S0007114515001415. PubMed PMID: 25990915.

2. Wang H, Xue Y, Zhang H, Huang Y, Yang G, Du M, et al. Dietary grape seed extract ameliorates symptoms of inflammatory bowel disease in IL10-deficient mice. Mol Nutr Food Res. 2013;57(12):2253-7. doi: 10.1002/mnfr.201300146. PubMed PMID: 23963706; PubMed Central PMCID: PMCPMC3976669.

3. Giulietti A, Overbergh L, Valckx D, Decallonne B, Bouillon R, Mathieu C. An overview of real-time quantitative PCR: applications to quantify cytokine gene expression. Methods. 2001;25(4):386-401. doi: 10.1006/meth.2001.1261. PubMed PMID: 11846608.

4. Cani PD, Bibiloni R, Knauf C, Waget A, Neyrinck AM, Delzenne NM, et al. Changes in gut microbiota control metabolic endotoxemia-induced inflammation in high-fat diet-induced obesity and diabetes in mice. Diabetes. 2008;57(6):1470-81. doi: 10.2337/db07-1403. PubMed PMID: 18305141.
