# Supplementary material for: Metformin Improves Ileal Epithelial Barrier Function in Interleukin-10 Deficient Mice
Source: PLoS One. 2016 Dec 21;11(12):e0168670. doi: 10.1371/journal.pone.0168670 (PMC5176295; doi:10.1371/journal.pone.0168670)
Supplement: S2 Table — (DOCX) [file pone.0168670.s002.docx]

**S2 Table. Primer sets used for quantitative RT-PCR in human cells**

| **Gene Name** | **Accession No.** | **Product Size** | **Direction** | **Sequence (5’🡪3’)** | **Source** |
| --- | --- | --- | --- | --- | --- |
| **β-actin** | NM_001101.3 | 100bp | Forward | GATGAGATTGGCATGGCTTT | This study |
|  |  |  | Reverse | CACCTTCACCGTTCCAGTTT |  |
| **BMP2** | NM_001200.2 | 177bp | Forward | TTCGGCCTGAAACAGAGACCCA | This study |
|  |  |  | Reverse | TTCCAAAGATTCTTCATGGTGGAAG |  |
| **BMP4** | NM_130851.2 | 172bp | Forward | GGAACATGGAGCCATTCCGT | This study |
|  |  |  | Reverse | ACGACCATCAGCATTCGGTT |  |
| **Math1** | NM_005172.1 | 154bp | Forward | TCGTTCAACAACGACAAGAAGCTGT | This study |
|  |  |  | Reverse | GAAGGTGGTGGTGGTCGCTTTT |  |
| **BMPR2** | NM_001204.6 | 234bp | Forward | TGGCAGCAGTATACAGATAGGTG | This study |
|  |  |  | Reverse | ATGGTTGTAGCAGTGCCTCC |  |
|  |  |  |  |  |  |
